# Simulation-Based Analysis of the Impact of Renal Impairment on the Pharmacokinetics of Highly Metabolized Compounds

**DOI:** 10.3390/pharmaceutics11030105

**Published:** 2019-03-02

**Authors:** Kristin E. Follman, Marilyn E. Morris

**Affiliations:** Department of Pharmaceutical Sciences, School of Pharmacy and Pharmaceutical Sciences, University at Buffalo, State University of New York, Buffalo, NY 14214, USA; kehill2@buffalo.edu

**Keywords:** renal impairment, drug metabolizing enzyme, protein binding, pharmacokinetics, simulation, physiologically based pharmacokinetic modeling, nifedipine, sildenafil, zidovudine

## Abstract

Renal impairment (RI) is a highly prevalent disease which can alter the pharmacokinetics (PK) of xenobiotics, including those that are predominately metabolized. The expression and activity of drug metabolizing enzymes (DMEs) and protein binding of compounds has been demonstrated to be affected in RI. A simulation based approach allows for the characterization of the impact of changes in these factors on the PK of compounds which are highly metabolized and allows for improved prediction of PK in RI. Simulations with physiologically based pharmacokinetic (PBPK) modeling was utilized to define the impact of these factors in PK in RI for a model substrate, nifedipine. Changes in fraction unbound and DME expression/activity had profound effects on PK in RI. Increasing fraction unbound and DME expression resulted in a reduction in exposure of nifedipine, while the reduction of DME activity resulted in an increase in exposure. In vitro and preclinical data were utilized to inform simulations for nifedipine, sildenafil and zidovudine. Increasing fraction unbound and changes in the expression/activity of DMEs led to improved predictions of PK. Further characterization of the impact of RI on these factors is warranted in order to better inform a priori predictions of PK in RI.

## 1. Introduction

Renal impairment (RI) is a major health concern for the United States and globally. RI is a serious disease due to its prevalence and mortality rate. Currently, RI affects 13.2% of adults over 30 years of age, and this has been projected to increase to 16.7% by 2030 [1]. RI carries an increased risk of mortality, specifically linked to cardiovascular function [2]. While a decrease in glomerular filtration rate (GFR) is a major defining characteristic of RI, the accumulation of uremic toxins in RI can also have a variety of effects. It has long been recognized that renal impairment has the potential to alter the pharmacokinetics (PK) of xenobiotics through alteration of renal clearance or protein binding of those compounds [3]. Recent studies have demonstrated the impact of RI can also extend to the expression and inhibition of both drug transporters and drug metabolizing enzymes (DMEs) [4,5,6,7,8,9]. 

The impact of RI on compounds that are mainly metabolized and have minimal renal clearance is of interest due to the potential alteration of DME expression and activity [6,7]. Two of the major DME superfamilies are the cytochrome P450s, (CYPs) and the UDP glucuronosyltransferases (UGTs). CYPs catalyze the oxidation of compounds and play a major role in Phase I metabolism [10]. UGTs represent a major Phase II metabolic pathway, responsible for the glucuronidation of compounds [11]. It has been demonstrated that the expression and activity of these enzymes can be altered in RI, or in the presence of uremic toxins which are present at elevated levels in RI populations [12,13,14,15,16]. Furthermore, the alteration of protein binding of compounds may also alter metabolic clearance of compounds [3,17,18]. Currently, it is recommended that PK studies are performed in an RI population for compounds which are likely to be administered to an RI population [19]. While clinical data in the desired population is the best avenue to obtain data to inform dosing currently, a simulation-based approach utilizing available in vivo and in vitro data may be useful to enhance the current mechanistic understanding of the impact of RI on PK.

Physiologically based pharmacokinetic (PBPK) modeling is an excellent tool to characterize the impact of RI on the PK of highly metabolized compounds. PBPK modeling uses physiological parameters and defines PK in a mechanistic manner, which allows the perturbation of different factors, such as protein binding and the activity of DMEs. By combining this platform with a simulation-based approach, model substrates can be utilized to define relationships between the factors affected in RI, such as free fraction and DME expression/activity, and PK parameters, such as area under the curve (AUC).

Simcyp^®^ is an excellent PBPK modeling platform which has several advantages for this study. First, the program contains renally impaired populations, including a moderate and severe RI population. These population files incorporate many physiological changes which are associated with RI. Simcyp^®^ also contains compound files, which are the result of previous modeling efforts which have been sufficiently verified to be included in the program for further use. Finally, Simcyp^®^ already includes some mechanistic changes which are relevant to metabolism in RI. Based on available clinical data, the expression of some of the CYP enzymes have been adjusted in the two renally impaired populations in the program. 

Nifedipine, sildenafil and zidovudine are all available in Simcyp^®^ and these compounds were chosen for this study. Nifedipine, a calcium channel blocker used for the treatment of cardiovascular disease, is a highly bound compound which is mainly eliminated by metabolism via CYP3A4 and CYP3A5 [20,21]. Based on these PK properties, this compound was selected as a model substrate to be utilized in a systematic evaluation of the effect of changes in protein binding, DME expression and activity on PK in RI. Sildenafil and zidovudine, a vasodilator and HIV antiviral, respectively, were included as model substrates to investigate the potential to improve predictions of PK in RI populations through the use of available preclinical and in vitro data. Sildenafil is metabolized by CYP3A4 and CYP2C9, while glucuronidation mediated by UGT2B7 is the major metabolic pathway for zidovudine [22,23]. The aims of this study were to utilize these model substrates to (1) investigate the impact of changes in protein binding and DME expression/activity on the PK of a model substrate; and (2) to explore the potential of further mechanistic modification based on available preclinical and in vitro data to improve predictions of PK in RI populations. 

## 2. Materials and Methods

### 2.1. Systematic Analysis

#### 2.1.1. Model and Software

Simulations were performed in Simcyp^®^ V17r1 using the built-in substrate file for nifedipine. The parameters for the nifedipine model are presented in Table S1. 

#### 2.1.2. Dose Dependency

The impact of dose dependency was investigated by performing simulations over the dose range of 10 to 180 mg orally. These were performed by varying the degree of RI by utilizing the following populations, Sim-Healthy Volunteers, Sim-RenalGFR_30-60, and Sim-RenalGFR_less_30 to represent 100%, 50% and 10% GFR. Simulations were performed separately in male and female populations, each simulation included 10 trials with 10 subjects in each. The area under the curve was calculated from the total plasma concentration time profiles for each substrate.

#### 2.1.3. CYP3A4 Expression

The impact of CYP3A4 expression was investigated by varying the expression of the enzyme ±2-, and ±5-fold. This range was selected to capture the potential impact of RI on expression. All simulations included a dose of 60 mg, with 10 trials with 10 subjects. Simulations were only conducted in a male population. The degree of RI was varied by utilizing the following populations, Sim-Healthy Volunteers, Sim-RenalGFR_30-60, and Sim-RenalGFR_less_30 to represent 100%, 50% and 10% GFR.

#### 2.1.4. Protein Binding

Fraction unbound was varied from the actual value of *f*_u_ for nifedipine (0.04) to 1.0 in male and female populations. Simulations were also conducted to investigate the impact of CYP3A4 expression with changes in *f*_u_. For these simulations, *f*_u_ was varied as described, and CYP3A4 expression was varied ±2-, and ±5-fold. All simulations included a dose of 60 mg, with 10 trials with 10 subjects in each, and were performed in healthy subjects and in the RI populations (Sim-Healthy Volunteers, Sim-RenalGFR_30-60, and Sim-RenalGFR_less_30 to represent 100%, 50% and 10% GFR).

#### 2.1.5. Competitive Inhibition

Inhibition was incorporated into the simulation through the use of the parameter *R*, which is defined in Equation (1),

(1) $$R=\frac{\left[I\right]}{K_{i}}$$

where *I* is the concentration of the inhibitor and *K*_i_ is the inhibition constant. *R* was then utilized to modify the activity of CYP3A4 in the nifedipine substrate file via Equation (2).

(2) $$v=\frac{V_{\max}}{K_{M}\left(1+R\right)+S}$$

*V*_max_ is the maximum rate of the metabolic reaction, *K*_M_ is the Michaelis-Menten affinity constant, and *S* is the substrate concentration. This equation allows for the incorporation of competitive inhibition, as *R* increases the apparent affinity of the enzyme for the substrate decreases. Although nifedipine is metabolized by both CYP3A4 and CYP3A5, inhibition was only included for CYP3A4, as this is the major route of elimination for the compound. *R* was varied from 0 to 100, simulations were performed separately in male and female populations. As with *f*_u_, the impact of CYP3A4 expression changes along with inhibition were investigated by varying enzyme expression ±2-, and ±5-fold. All simulations included a dose of 60 mg, 10 trials with 10 subjects in each and were performed in healthy subjects and in the RI populations (Sim-Healthy Volunteers, Sim-RenalGFR_30-60, and Sim-RenalGFR_less_30 to represent 100%, 50% and 10% GFR).

#### 2.1.6. Protein Binding and Competitive Inhibition

Simulations were performed varying *f*_u_ (0.04 to 1.0) and R (0 to 100) to investigate the impact of competitive inhibition with concurrent changes in protein binding. All simulations included a dose of 60 mg, with 10 trials with 10 subjects in each, and were performed in healthy subjects and in the RI populations (Sim-Healthy Volunteers, Sim-RenalGFR_30-60, and Sim-RenalGFR_less_30 to represent 100%, 50% and 10% GFR.

#### 2.1.7. Clinical Comparison

Model substrates were utilized in order to assess the impact of including changes in factors including DME expression, *f*_u_, and DME inhibition on the prediction of clinical PK in renally impaired populations. In addition to nifedipine, sildenafil and zidovudine were selected; models for these compounds were selected from the built-in compounds available in Simcyp^®^ V17r1. 

All three compounds, nifedipine, sildenafil and zidovudine exhibit plasma protein binding (*f*_u_ of 0.039, 0.036 and 0.80, respectively); therefore, the impact of RI on *f*_u_ was included. Literature reports of changes in protein binding in RI indicate increases of *f*_u_ of up to 54% (Table S2) [18,24]. Simulations included increases in *f*_u_ of 5, 10 and 50% for nifedipine and sildenafil. For zidovudine, *f*_u_ was increased 5 and 10% and was also set to its maximal value of 1.0.

Data for clinical comparisons were obtained from literature references which contained either PK parameters for the compound of interest in an RI population, or PK parameters and concentration time profiles for the compound. For nifedipine, only PK parameters were available for the conditions investigated (van Bortel et al. [25]). The data for sildenafil and zidovudine were obtained from Muirhead et al. [26] and Pioger et al. [27]. Data for moderate RI included the mild and moderate RI populations for sildenafil, and the comparison for PK parameters was made to an average of the PK parameters for these two populations [26].

### 2.2. Incorporation of Enzyme Inhibition

#### 2.2.1. CYP3A 

Nifedipine and sildenafil are substrates of CYP3A4. Nifedipine is also a substrate of CYP3A5. Inhibition of CYP3A4 was also included for these compounds. Inhibition was incorporated as it was in the systematic simulations, through the use of the factor *R* (Equation (1)). In order to obtain relevant *R* values for these simulations, the impact of inhibition of uremic toxins on CYP3A4 activity was obtained from the literature. Benzyl alcohol, indoxyl sulfate and p-cresol are toxins which can accumulate in RI, and these compounds have been shown to inhibit CYP3A4 activity to varying degrees [14]. Based on approximate IC_50_ values, and their concentration in RI populations, it was determined that *R* for CYP3A4 could vary between 1 and 26.6 (Table S3) [16]. Therefore, simulations were performed for nifedipine and sildenafil including R values of 1 and 10 for CYP3A4.

#### 2.2.2. CYP2C9 

Sildenafil is also a substrate of CYP2C9. CYP2C9 has also been shown to be inhibited by various uremic toxins, including *p*-cresol and hippuric acid [14]. Again, based on the affinity of these inhibitors and their concentration in RI populations, the maximal anticipated *R* for CYP2C9 in RI would be 2.4 (Table S3) [16]. Simulations for sildenafil included *R* values of 1 and 10. 

#### 2.2.3. UGT2B7

Zidovudine is mainly eliminated via UGT2B7, and this enzyme is also inhibited by uremic toxins including hippuric acid, indoxyl sulfate and *p*-cresol [14]. For this enzyme, the maximal anticipated *R* in an RI population was 2.5 (Table S3). Simulations for zidovudine included *R* values for UGT2B7 of 1 and 10. 

Every possible combination of these factors for each compound were simulated using the demographics of the clinical study for comparison. The moderate and severe RI populations in Simcyp^®^ include some modifications to CYP expression in the liver. These modifications include reducing the CYP expression. In order to investigate the impact of this modified CYP expression on the clinical comparison, two sets of simulations were performed for nifedipine and sildenafil, the two compounds that would be affected by changes in CYP expression. One set of simulations utilized the modified expression present in the renal population, another set used the unmodified expression found in the healthy subject population. 

Simulations were then ranked using the parameter *δ* (Equation (3)), which takes the absolute value of the log of the predicted parameter over the observed parameter. The sum of the *δ* values for each parameter (Σ*δ*_all_) was used to rank the simulations against each other, with Σ*δ*_all_ values closest to 0 representing the best simulations.

(3) $$\delta =\left|log\left(\frac{predicted}{observed}\right)\right|$$

Once the best simulations were identified, these simulations were compared to the simulations using the base RI population and base substrate files. The ratio of the predicted parameter and the observed parameter were compared to see if the modified simulations identified by Σ*δ*_all_ improved the majority of the parameter predictions. The predicted plasma profiles were also plotted along with the base file predictions. 

## 3. Results

### 3.1. Systematic Analysis with Nifedipine as the Model Substrate

#### 3.1.1. Dose Dependency

**Figure 1:**
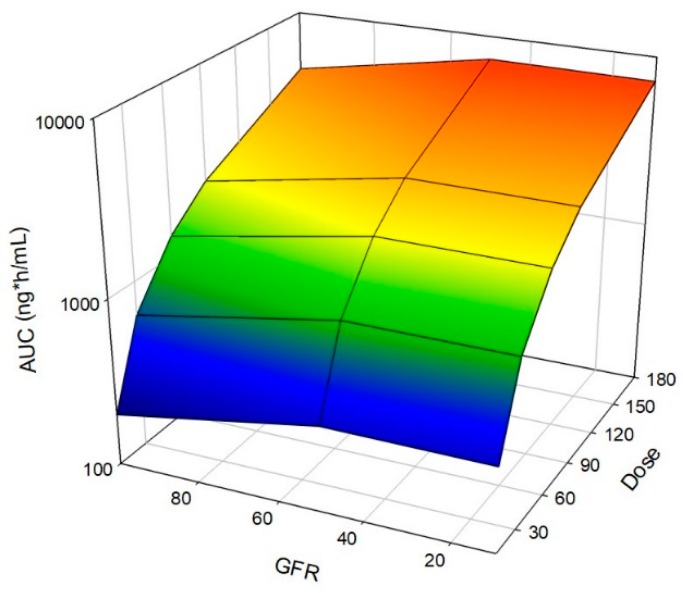
Impact of Renal Impairment on AUC of Nifedipine. Simulations with increasing oral doses (30 to 180 mg) were administered in a fasted state to an exclusively male population. Simulations included ten trials with ten individuals in each trial. Age ranged from 21 to 65 years, and simulations were conducted with varying the degrees of RI by utilizing the following populations, Sim-Healthy Volunteers, Sim-RenalGFR_30-60, and Sim-RenalGFR_less_30 to represent 100%, 50% and 10% GFR.

For each dose, AUC increased approximately 1.6 with decreasing GFR for males in both moderate and severe populations (Figure 1, Table S4). The impact of reducing GFR increased with increasing dose, but this increase was marginal (Table S4). Trends for females were identical to males, but the absolute value for exposure was reduced (Table S4). Data for simulations in female populations are provided in the supplementary information. Figures S1–S3 depict the impact of GFR, *f*_u_ and inhibition on AUC for females. AUC values are provided along with the data for males, for comparison, in Tables S4, S6 and S9. 

#### 3.1.2. CYP3A4 Expression

**Figure 2:**
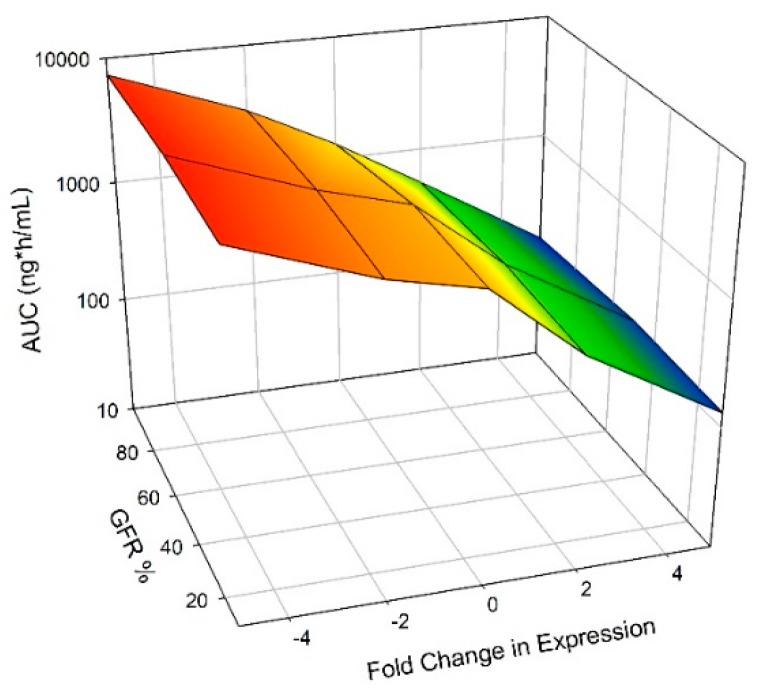
Impact of CYP3A4 Expression on AUC of Nifedipine. CYP3A4 expression was varied ±2- and ±5-fold. A dose of 60 mg was administered orally to an exclusively male population in a fasted state. Simulations included ten trials with ten individuals in each. Age ranged from 21 to 65 years, and simulations were conducted with varying the degrees of RI by utilizing the following populations, Sim-Healthy Volunteers, Sim-RenalGFR_30-60, and Sim-RenalGFR_less_30 to represent 100%, 50% and 10% GFR.

As CYP3A4 expression was increased, AUC decreased and as CYP3A4 expression was decreased, AUC increased (Figure 2). For healthy individuals, AUC decreased 2.6-, and 11-fold when CYP3A4 expression was increased +2-, and +5-fold, respectively (Figure 2, Table S5). This was magnified in RI, AUC decreased 3.6-, and 15-fold for moderate RI and 3.9-, and 16-fold for severe RI (Figure 2, Table S5). AUC increased 2.3-, and 5.6-fold when CYP3A4 expression was decreased 2-, and 5-fold, respectively, for healthy individuals (Figure 2, Table S5). This was minimized in RI, 1.6-, and 3.8-fold for moderate RI, and 1.4-, and 3.5-fold for severe RI for −2-, and −5-fold CYP3A4 expression, respectively (Figure 2, Table S5).

#### 3.1.3. Protein Binding

**Figure 3:**
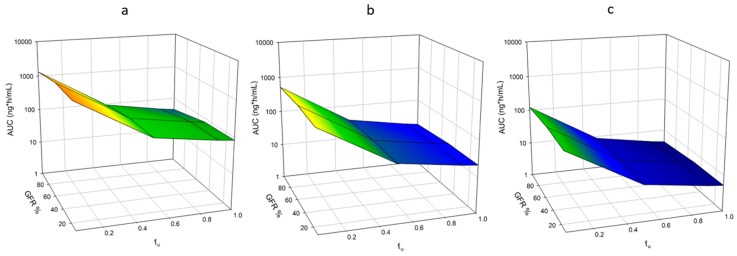

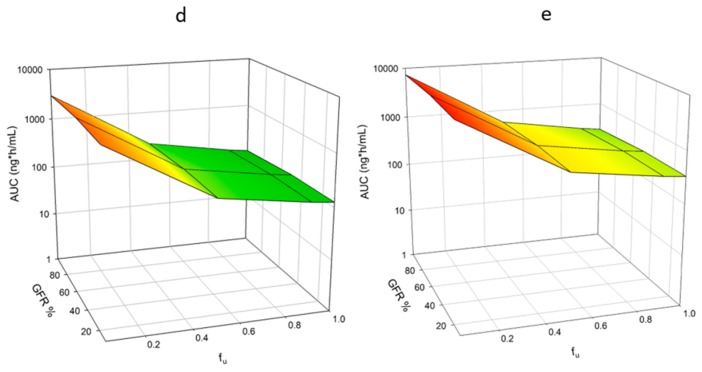
Impact of Fraction Unbound and Enzyme Expression on AUC of Nifedipine. Simulations were performed varying fraction unbound (*f*_u_) from the observed value for nifedipine (0.04) to 1.0. Expression of CYP3A4 was also varied ±2- and ±5-fold. A dose of 60 mg was administered orally to an exclusively male population in a fasted state. Simulations included ten trials with ten individuals in each. Age ranged from 21 to 65 years, and simulations were conducted with varying the degrees of RI by utilizing the following populations, Sim-Healthy Volunteers, Sim-RenalGFR_30-60, and Sim-RenalGFR_less_30 to represent 100%, 50% and 10% GFR. (**a**) Original CYP3A4 expression; (**b**) +2-fold CYP3A4 expression; (**c**) +5-fold CYP3A4 expression; (**d**) −2-fold CYP3A4 expression; (**e**) −5-fold CYP3A4 expression.

AUC decreased as *f*_u_ increased. AUC was reduced from 1.32 × 10^3^ to 43.7 ng·h/mL when *f*_u_ was increased from 0.04 to 1.0 in healthy males, a reduction of 30-fold (Figure 3a, Table S6). This impact of *f*_u_ was slightly reduced as renal function declined, a reduction of 28- and 25-fold was observed for moderate and severe RI populations (Figure 3a, Table S6). 

Changes in protein binding also had an impact on total oral clearance (CL/F) and volume of distribution (*V*_ss_). When *f*_u_ was increased both CL/F and *V*_ss_ were also increased (Table S7). The changes in *V*_ss_ were not altered with the incorporation of RI, while the increase in CL/F was minimized as renal function declined (Table S7).

#### 3.1.4. Protein Binding and CYP3A4 Expression

As CYP3A4 expression was increased, the fold decrease in AUC when *f*_u_ was increased from 0.04 to 1.0 were identical to the original expression for healthy and RI populations (Figure 3a, Table S8). As expression was decreased, the impact of *f*_u_ was minimized. AUC decreased 30-, 29-, and 27-fold for original, +2-, and +5-fold CYP3A4 expression, respectively (Figure 3b,c, Table S8). The impact of *f*_u_ was minimized when RI was included. AUC decreased 27-, and 24-fold for moderate RI, and 24-, and 22-fold for severe RI for −2-, and −5-fold CYP3A4 expression, respectively (Figure 3d,e, Table S8).

#### 3.1.5. Competitive Inhibition

**Figure 4:**
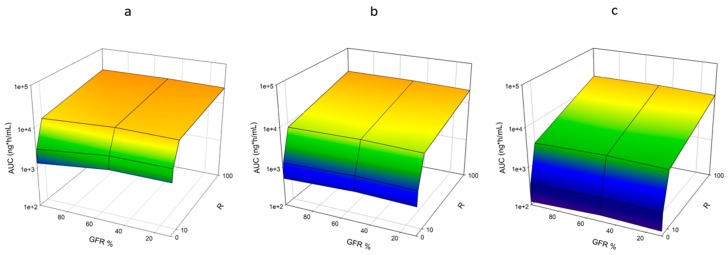

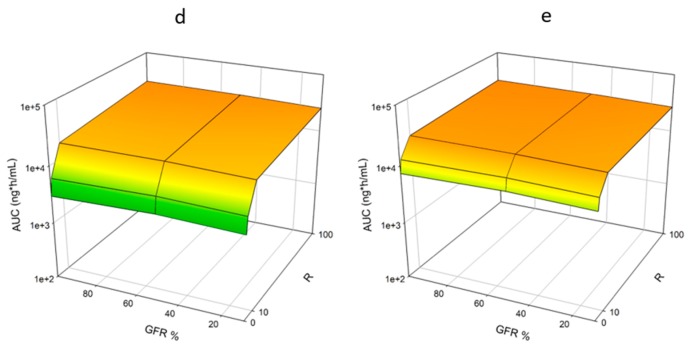
Impact of Competitive Inhibition and Enzyme Expression on AUC of Nifedipine. Simulations were performed incorporating competitive inhibition through the factor R ([I]/*K*_i_). R was varied from 0 to 100. Expression of CYP3A4 was also varied ±2- and ±5-fold. A dose of 60 mg was administered orally to an exclusively male population in a fasted state. Simulations included ten trials with ten individuals in each. Age ranged from 21 to 65 years, and simulations were conducted with varying the degrees of RI by utilizing the following populations, Sim-Healthy Volunteers, Sim-RenalGFR_30-60, and Sim-RenalGFR_less_30 to represent 100%, 50% and 10% GFR. (**a**) Original CYP3A4 expression; (**b**) +2-fold CYP3A4 expression; (**c**) +5-fold CYP3A4 expression; (**d**) −2-fold CYP3A4 expression; (**e**) −5-fold CYP3A4 expression.

As competitive inhibition was increased for CYP3A4, AUC was increased for nifedipine. When *R* was increased from 0 (no inhibition) to 100 (maximal inhibition investigated) AUC increased 20-fold for healthy male individuals (Figure 4a, Table S9). The effect was reduced as RI was introduced. AUC increased 13- and 12-fold for males with moderate and severe RI, respectively (Figure 4a, Table S9). 

#### 3.1.6. Competitive Inhibition and CYP3A4 Expression

The impact of varying CYP3A4 expression was investigated in males by varying the expression of CYP3A4 ±2-, and ±5-fold. As CYP3A4 expression was increased, the impact of inhibition was magnified. For healthy individuals, AUC increased 20-, 46-, and 143-fold for original, +2- and +5-fold CYP3A4 expression, respectively (Figure 4a–c, Table S10). Regardless of expression, the presence of RI reduced the effect of inhibition on AUC. AUC increased 39-, and 120-fold for moderate RI, and 39-, and 119-fold for severe RI for −2-, and −5-fold, respectively (Figure 4b,c, Table S10). 

When CYP3A4 expression was decreased, the impact of inhibition was minimized. AUC only increased 9.7- and 4.1-fold for −2-, and −5-fold CYP3A4 expression, respectively, compared with the 20-fold increase observed with original enzyme expression (Figure 4a,d,e, Table S10). Again, the presence of RI minimized the effect of inhibition regardless of changes in enzyme expression. AUC increased 8.3-, and 3.6-fold for moderate RI, and 8.3-, and 3.7-fold for severe RI for −2-, and −5-fold expression, respectively (Figure 4d,e, Table S10). 

#### 3.1.7. Protein Binding and Competitive Inhibition

**Figure 5:**
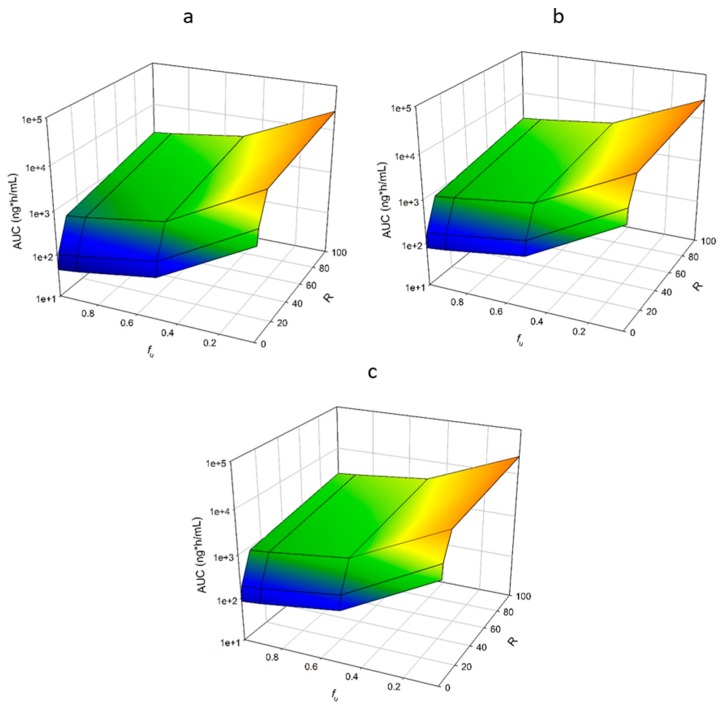
Impact of Fraction Unbound and Competitive Inhibition on AUC of Nifedipine. The impact of concurrent changes in *f*_u_ and competitive inhibition was investigated. *f*_u_ was varied from 0.04 to 1.0, and the factor R ([I]/*K*_i_) was varied from 0 to 100. Age ranged from 21 to 65 years, and simulations were conducted with varying the degrees of RI by utilizing the following populations, (**a**) Sim-Healthy Volunteers; (**b**) Sim-RenalGFR_30-60; (**c**) Sim-RenalGFR_less_30.

Competitive inhibition increased AUC, and this effect was magnified as *f*_u_ was increased. When *R* was increased from 0 to 100 with the original *f*_u_ for nifedipine (0.04), AUC increased 20-fold for healthy individuals (Figure 5a, Table S11). As *f*_u_ increased to 1.0 with the same increase in *R* (0 to 100), AUC was increased 45-fold (Figure 5a, Table S11). The same trend was observed when RI was incorporated, but the magnitude of the changes was reduced. For moderate RI, when *R* was increased from 0 to 100, AUC increased 13-, and 30-fold for a *f*_u_ of 0.04 and 1.0, respectively (Figure 5b, Table S11). For severe RI, for the same increase in RI, AUC increased 12-, and 27-fold for a *f*_u_ of 0.04 and 1.0, respectively (Figure 5c, Table S11).

### 3.2. Clinical Comparison

For nifedipine, 30 simulations with modified CYP3A4 activity and/or protein binding were performed. The simulation which provided the lowest Σ*δ*_all_ with the modified CYP expression which is present in the Simcyp^®^ severe renal impairment population was the simulation run with a 5% increase in *f*_u_ for nifedipine (Table 1). For this simulation, there was an improvement in the prediction of maximum concentration (*C*_max_), AUC and oral clearance (clearance/bioavailability or CL/F) (Table 1). The most improved simulation (lowest Σ*δ*_all_) with unmodified CYP expression also included an increase in *f*_u_ (10%) but also included inhibition of CYP3A4 with competitive inhibition with an R of 1 (Table 1). Both of these simulations included an improvement in the predicted/observed ratio for *C*_max_, AUC and CL/F (Table 1). The base simulation, utilizing the unmodified Simcyp^®^ severe RI population and nifedipine files, gave the most accurate prediction of *t*_max_ and F (Table 1). Simulations are not plotted with observed data as concentration time profiles were not provided in the literature reference for a single oral dose of nifedipine. The comparison is restricted to the reported PK parameters for these conditions.

There were 126 modified simulations performed for sildenafil. The best simulation with the modified CYP expression which is present in the Simcyp^®^ severe renal impairment population, as indicated by Σ*δ*_all_, included a 50% increase in *f*_u_ for sildenafil, as well as competitive inhibition of CYP3A4 with an R value of 10. With these modifications, there was an improvement over the base prediction for *t*_max_, AUC and CL/F for sildenafil, but not for *C*_max_ (Table 2).

**Figure 6:**
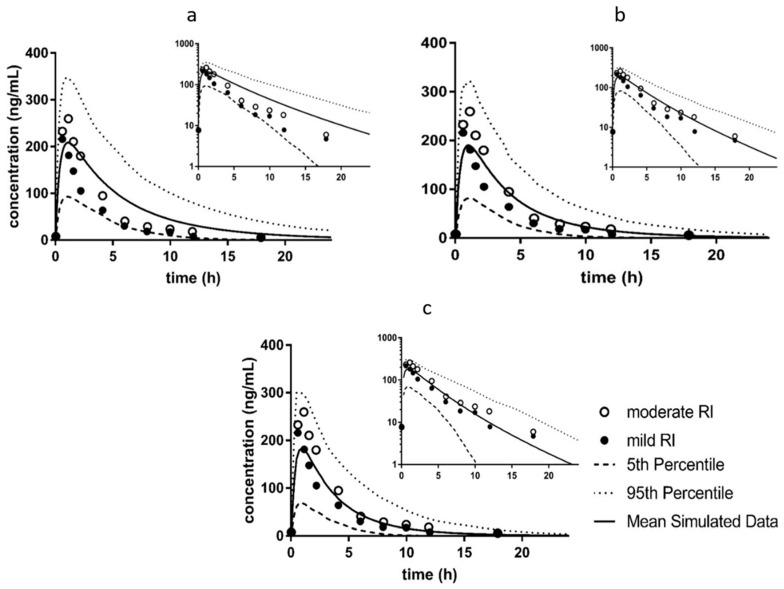
Comparison of Predicted Plasma Profiles for Sildenafil in Renally Impaired Patients for Base and Modified Simulations. Simulations were performed with oral administration of 50 mg of sildenafil to men in a fasted state, age 18–70, the duration of the simulation was 48 h. These conditions are consistent with the parameters of the clinical study from which the observed data was obtained [26]. Simulations were performed with ten trials with ten subjects in each trial. Graphs represent that data on a linear scale, with the inset graphs representing the same data on a log scale. Open circles are observed mild RI data (GFR 50–80 mL/min), closed circles are observed moderate RI data (GFR 30–50 mL/min), solid lines are the mean of the simulation, the dotted line is the 95^th^ percentile, and the dashed lines are the 5^th^ percentile. (**a**) Base Simulation—Sim GFR 30_60—simulation performed with the base moderate RI population and base sildenafil compound file; (**b**) Modified Simulation with original CYP expression—*f*_u_ +5% CYP2C9 R1 CYP3A4 R1—simulation performed with a 5% increase in f_u_ for sildenafil, and competitive inhibition of CYP2C9 and CYP3A4 with a R ([I]/*K*_i_) values of 1 for both enzymes; (**c**) Modified Simulation with modified CYP expression—*f*_u_ +50% CYP3A4 R10 —simulation performed with a 50% increase in *f*_u_ for sildenafil and competitive inhibition of CYP3A4 with a R (I/[*K*_i_]) value of 10.

The prediction for *C*_max_ was similar between the base and modified simulations (Table 2). For simulations which included modified CYP expression included in the RI populations in Simcyp^®^, the most improved simulation based on Σ*δ*_all_ included an increase in *f*_u_ (5%) and inhibition of CYP3A4 and CYP2C9 with an *R* of 1 for both enzymes (Table 2). Figure 6 shows the plasma profile predictions for sildenafil for the base (Figure 6a) and modified (Figure 6b,c) simulations. These profiles show that while the *C*_max_ is not as well captured in the modified simulation, there is an improvement in the terminal phase prediction (Figure 6).

**Figure 7:**
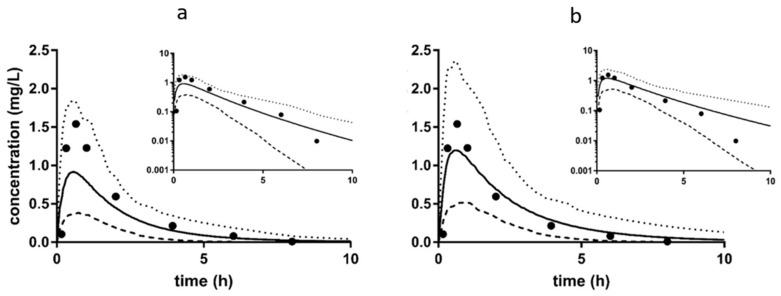
Comparison of Predicted Plasma Profiles for Zidovudine in Renally Impaired Patients for Base and Modified Simulations. Simulations were performed with oral administration of 200 mg of sildenafil to subjects (proportion of females 0.428) in a fasted state, age 56–62, the duration of the simulation was 48 h. These conditions are consistent with the parameters of the clinical study from which the observed data was obtained [27]. Simulations were performed with ten trials with ten subjects in each trial. Graphs represent that data on a linear scale, with the inset graphs representing the same data on a log scale. Closed circles are observed data, solid lines are the mean of the simulation, the dotted line is the 95^th^ percentile, and the dashed lines are the 5^th^ percentile. (**a**) Base Simulation—Sim GFR less than 30—simulation performed with the base moderate RI population and base zidovudine compound file; (**b**) Modified Simulation—*f*_u_ +25% UGT2B7 R1—simulation performed with a 25% increase in *f*_u_ for zidovudine, and competitive inhibition of UGT2B7 with an R ([I]/*K*_i_) value of 1.

For zidovudine, only 15 modified simulations were necessary to incorporate all of the changes in *f*_u_ and UGT2B7 inhibition that were indicated by the literature. As CYP metabolism is not included in the model for zidovudine, only one set of simulations were performed. Of these, the best simulation as predicted by Σ*δ*_all_ included a 25% increase in *f*_u_ for zidovudine, as well as competitive inhibition of UGT2B7 with an R value of 1. With these modifications, there was an improvement in the predictions for *C*_max_, *t*_max_, AUC and CL/F when compared to the predictions for the base simulation (Table 3). The plasma profiles for the base simulation (Figure 7a) and the best modified simulation (Figure 7b) show the improvement in the prediction of *C*_max_ for the modified simulation.

## 4. Discussion

Renal impairment is a major health concern worldwide, and has a high prevalence in the United States [1]. RI has been demonstrated to result in altered PK for compounds, including those that are mainly eliminated via metabolism and experience negligible renal clearance [5,7,12,25,26,27]. The effect of RI on such compounds is likely due to changes in DME expression, activity, protein binding, or a combination of these factors. Uremic toxins, compounds which accumulate in RI due to their decreased clearance, have been shown to alter DME expression and inhibit their activity [12,13,14,15,16]. These toxins may also affect the protein binding of drugs by competing for binding with proteins [17,18,24]. Additionally, physiological changes, such as hypoalbuminemia, or pH changes may further impair protein binding of compounds in RI [18]. In order to further define and understand the potential implication of RI to mechanistically impact PK of highly metabolized compounds, a simulation-based approach utilizing PBPK modeling was utilized in this investigation.

Incorporating a reduction in GFR into simulations of nifedipine PK resulted in an increase in exposure for the compound and the effect was magnified as dose increased. This was expected with RI, as the condition typically leads to a lower overall clearance and therefore a higher AUC. Increasing DME expression resulted in a decrease in AUC for nifedipine; this is expected, as the increase in expression of DMEs results in an increased capacity for the system to metabolize the compound. Similarly, as DME expression was decreased, AUC was increased as the capacity to clear nifedipine was reduced. As metabolism is the main route of elimination, the effect of changing DME expression was greater than the inclusion of RI alone.

Changes in fraction unbound also had a large effect on AUC. When fraction unbound was increased, as can be expected in RI for many drugs, exposure was decreased. The increased free fraction of the compound for metabolism resulted in increased clearance and a reduction in AUC. Interestingly, the effect was minimized as renal function declined. As declining renal function results in elimination that is more heavily dependent on metabolism, and increasing *f*_u_ results in more substrate available for metabolism, this may lead to a saturation of this route of elimination, therefore reducing the effect of fraction unbound. The impact of *f*_u_ was also reduced when DME expression was increased, and this was not altered by a decline in renal function, which is consistent with expectation for high hepatic CL compounds. The increase in AUC observed with an increase in *f*_u_ from 0.04, the observed value for nifedipine, to 1.0, was consistent despite an increase in DME expression. However, when DME expression was decreased, the impact of *f*_u_ on AUC was reduced. These results may support the idea that the ratio of available substrate to the capacity of the enzyme is what drives changes in exposure. Since RI has the potential to alter the capacity (expression) of DMEs, and the availability of the substrate, it may be important to understand how protein binding will be affected for a substrate of interest, and how relevant enzymes may be affected in RI. This may also be explained by examining the well-stirred model of hepatic clearance, which is utilized in these simulations. As DME expression increases, intrinsic clearance (CL_int_) also increases, as this is the ratio of *V*_max_ to *K*_M_. As DME expression was increased, there was a decrease in AUC, as expected, but when *f*_u_ was also increased, the change in AUC was consistent regardless of an increase in DME expression. This could be due to the increase in *f*_u_ being more influential than change in expression, as CL_int_ is high compared to hepatic blood flow and hepatic clearance is reaching a maximum value which is limited by hepatic blood flow. Conversely, as DME expression decreased, the decrease in AUC driven by an increase in *f*_u_ was minimized, meaning that hepatic clearance was altered by both changes in *f*_u_ and DME expression. This is because hepatic clearance was not approaching hepatic blood flow, but CL_int_ and *f*_u_ were still contributing to the observed changes in AUC.

As expected, CL/F and *V*_ss_ both increased with increasing *f*_u_. This is due to the higher availability of the free compound for elimination, mainly via metabolism, and for distribution to tissues. The changes in *V*_ss_ were not substantially altered in the presence of RI, indicating that the enhanced distribution is mainly dependent on the value of *f*_u_, and not affected by other parameters altered in RI. The increase in CL/F was minimized as renal function declined, while the higher free fraction allowed for enhanced elimination which was reduced in the presence of RI. This is likely due to the reduced enzyme expression in the RI populations in Simcyp.

Incorporating competitive inhibition of DMEs resulted in an expected increase in AUC. When DME expression was increased, the impact of inhibition was magnified; conversely, when DME expression was decreased the impact of inhibition was minimized. Regardless of any changes in DME expression, the impact of inhibition was minimized as renal function declined. The reduced effect in the presence of RI may be related to the availability of the substrate overall. As RI is incorporated, there is an increase in exposure of the substrate that may minimize the effect of a competitive inhibitor. The ratio of substrate to inhibitor is different for a healthy individual compared to a renally impaired individual, resulting in a smaller effect of inhibition. When *f*_u_ was increased along with inhibition, the effect of inhibition on AUC was magnified, due to changes in hepatic clearance. These effects were minimized in RI, due to changes in hepatic clearance, which were affected by both changes in CL_int_, due to inhibition, and *f*_u_. In the RI population in Simcyp, there is a reduction in the hepatic expression of CYP enzymes. As the RI population already has a reduced CL_int_, due to a reduced expression in RI of CYP enzymes, the impact of inhibition and *f*_u_ changes are small.

PBPK modeling and simulation has been increasingly utilized for the prediction and characterization of PK in special populations [28,29,30,31,32]. The simulation-based approach utilized in this work, combined with PBPK modeling provides insight to the relationships of factors such as protein binding and DME expression/activity with the exposure of a highly metabolized substrate. Further understanding of these relationships and defining of changes in these factors which may be affected in RI will provide more accurate prediction of changes in PK for highly metabolized substrates. In order to evaluate the utility of a simulation-based approach to validate in vitro or preclinical data for changes in factors such as DME activity and fraction unbound, simulations were performed with the base Simcyp^®^ populations and compound files with and without modification to determine if modification could result in improved predictions. A literature search was conducted to understand what degree of inhibition and changes in protein binding could reasonably be expected in RI. R values ([I]/*K*_i_) were determined utilizing in vitro potency values for individual uremic toxins against DMEs which were relevant for the model substrates and concentrations of uremic toxins which have been observed in vivo. Similarly, changes in protein binding have been observed up to 50%, and so increases in *f*_u_ of 5, 10 and 50% were utilized in simulations.

This work provides a proof of concept for utilization of in vitro data, preclinical data, and a simulation based approach for incorporating changes in DME activity and protein binding for improvement of prediction of PK in a RI population. The impact of various factors on exposure for highly metabolized compounds was characterized both qualitatively and quantitatively, which enables a more complete understanding of the impact of RI on PK. Overall, the systematic simulation investigation with nifedipine demonstrated that changes in the fraction unbound and DME expression/activity represent the major determinants of altered PK in RI. As expected, increasing fraction unbound and DME expression resulted in a reduction in exposure of nifedipine, while the reduction of DME activity resulted in an increase in exposure. The relationships between these various factors in RI and their impact on the PK of nifedipine provides a framework for discussion of the potential changes in PK for RI populations for other highly metabolized compounds. It was determined that modification of these parameters with readily available information in the literature did lead to improvement of prediction of PK for all three substrates, nifedipine, sildenafil and zidovudine. Modifications included inhibition of DMEs, increases in *f*_u_ and the modification of DME expression which is present in the RI population files in Simcyp^®^. Interestingly, improvements were made without the modification to DME expression as well; typically these improvements were observed with a higher degree of inhibition for DMEs. The improved ability of the modified simulations to capture the PK in RI populations highlights the need to incorporate changes in DME expression/activity and protein binding in RI. The results also indicate the need for further investigation of the particular changes which can occur within RI, as PK predictions were improved to a similar degree for nifedipine and sildenafil with or without modification of CYP expression. It may be possible to identify the changes which are truly occurring in RI with more in vitro data, or more detailed clinical data. There are limitations to obtaining this information. It is very costly to perform clinical studies, and in vitro data may not accurately represent the in vivo situation [33]. The inclusion of more model substrates with similar pathways of elimination, may decrease the need for extensive clinical data collection. Additionally, inclusion of more substrates may reduce the number of conditions which lead to improvement of PK for each substrate, and this process may allow more confidence in the extrapolation of results to a priori predictions of PK in RI populations for highly metabolized compounds. Providing more accurate predictions of PK may enable a reduction in the number and size of clinical trials which are required to adequately inform any necessary dosing adjustments in RI populations.

## Figures and Tables

**Table 1:** Comparison of Predicted and Observed Values for Pharmacokinetic Parameters for Nifedipine in Severely Renally Impaired Patients.

PK Parameter	*C*_max_ ng/mL	*t*_max_ h	AUC ng*h/mL	CL/F L/h	F	Σ*δ*_all_
Observed [25]	75.0	0.500	102	138	0.400	
Sim GFR less than 30	119	0.340	274	43.6	0.45	1.35
predicted/observed	1.59	0.680	2.69	0.316	1.11	
*f*_u_ +10% CYP3A4 R1 (unmodified expression)	60.9	0.212	111	96.0	0.377	0.697
predicted/observed	**0.811**	**0.424**	**1.09**	**0.695**	**0.942**	
*f*_u_ +5% (modified expression)	64.5	0.236	121	108	0.345	0.648
predicted/observed	**0.859**	0.471	**1.18**	**0.782**	0.863	

Notes: Bolded values indicate an improvement in predicted/observed values for the modified simulations compared to the simulation using the base population and compound files. Sim GFR less than 30—simulation performed with the base severe RI population and base nifedipine compound file. *f*_u_ +10% CYP3A4 R1—simulation performed with a 10% increase in *f*_u_ for nifedipine, and competitive inhibition of CYP3A4 with a *R* ([I]/*K*_i_) value of 1. *C*_max_—Maximum concentration; *t*_max_—Time to maximum concentration; AUC—Area under the curve; CL/F—Clearance over the bioavailability; F—Bioavailability.

**Table 2:** Comparison of Predicted and Observed Values for Pharmacokinetic Parameters for Sildenafil in Moderately Renally Impaired Patients.

PK Parameter	*C*_max_ ng/mL	*t*_max_ h	AUC ng*h/mL	CL/F mL/min	Σδ_all_
Observed [26]	272	0.750	783	65.0	
Sim GFR 30_60	212	1.16	1440	44.4	0.729
predicted/observed	0.780	1.55	1.84	0.680	
*f*_u_ +5% CYP2C9 R1 CYP3A4 R1 (unmodified expression)	193	1.04	983	64.6	0.395
predicted/observed	b	**1.39**	**1.26**	**0.99**	
*f*_u_ +50% CYP3A4 R10 (modified expression)	186	0.980	831	79.2	0.393
predicted/observed	0.684	**1.31**	**1.06**	**1.22**	

Notes: Bolded values indicate an improvement in predicted/observed values for the modified simulations compared to the simulation using the base population and compound files. Sim GFR 30_60—simulation performed with the base moderate RI population and base sildenafil compound file. *f*_u_ +5% CYP2C9 R1 CYP3A4 R1—simulation performed with a 5% increase in *f*_u_ for sildenafil, and competitive inhibition of CYP2C9 and CYP3A4 with a R ([I]/*K*_i_) values of 1 for both enzymes.

**Table 3:** Comparison of Predicted and Observed Values for Pharmacokinetic Parameters for Zidovudine in Severely Renally Impaired Patients.

PK Parameter	*C*_max_ µmol/L	*t*_max_ h	AUC µmol*h/L	CL/F L/h	Σ*δ*_all_
Observed [27]	6.2	0.7	11.7	73.7	
Sim GFR less than 30	3.49	0.59	8.32	111	1.71
predicted/observed	0.56	0.84	0.71	1.51	
fu +25% UGT2B7 R1	4.57	0.65	13.3	68.9	1.42
predicted/observed	**0.74**	**0.92**	**1.13**	**0.93**	

Notes: Bolded values indicate an improvement in predicted/observed values for the modified simulations compared to the simulation using the base population and compound files. Sim GFR less than 30—simulation performed with the base severe RI population and base zidovudine compound file. *f*_u_ +25% UGT2B7 R1—simulation performed with a 25% increase in *f*_u_ for zidovudine, and competitive inhibition of UGT2B7 with a R ([I]/*K*_i_) value of 1.

## Supplementary Materials

The following are available online at https://www.mdpi.com/1999-4923/11/3/105/s1, Table S1: Parameters for Nifedipine Model; Table S2: Impact of Renal Impairment on Fraction Unbound; Table S3: Approximation of R Values for Simulation; Table S4: Impact of Renal Impairment on AUC in Males and Females; Table S5: Impact of CYP3A4 Expression on AUC; Table S6: Impact of Fraction Unbound on AUC for Males and Females; Table S7: Impact of Changes in Fraction Unbound on Oral Clearance and Volume of Distribution in Males; Table S8: Impact of Fraction Unbound on AUC with Changes in CYP3A4 Expression; Table S9: Impact of Competitive Inhibition on AUC in Males and Females; Table S10: Impact of Competitive Inhibition and CYP3A4 Expression on AUC in Males; Table S11: Impact of Fraction Unbound and Competitive Inhibition on AUC; Figure S1: Impact of Renal Impairment on AUC in Females; Figure S2: Impact of Fraction Unbound on AUC in Females; Figure S3: Impact of Competitive Inhibition on AUC in Females.
