# Supplementary material for: Simulation-Based Analysis of the Impact of Renal Impairment on the Pharmacokinetics of Highly Metabolized Compounds
Source: Pharmaceutics. 2019 Mar 2;11(3):105. doi: 10.3390/pharmaceutics11030105 (PMC6471170; doi:10.3390/pharmaceutics11030105)
Supplement: Supplementary file 1 [file pharmaceutics-11-00105-s001.pdf]

pharmaceutics

Supplementary Materials: Simulation-Based Analysis of the Impact of Renal Impairment on the Pharmacokinetics of Highly Metabolized Compounds

Kristin E. Follman and Marilyn E. Morris *

Table S1. Parameters for Nifedipine Model.

**PhysChem and Blood Binding**	
Mol Weight (g/mol)	346.300
log P	2.690
Compound Type	Monoprotic Base
pKa 1	2.390
B/P	0.740
fu	0.040
**Absorption**	
Absorption Model	1st order
fa (predicted)	0.996
ka (1/h) (predicted)	2.455
Peff,man (10-4 cm/s) (predicted)	5.621
**Distribution**	
Distribution Model	Full PBPK Model
Vss (L/kg) (predicted)	0.644
**Elimination**	
CYP3A4	
Vmax (pmol/min/pmol)	17.880
Km (μM)	9.110
fu mic	1.000
CYP3A5	
Vmax (pmol/min/pmol)	23.050
Km (μM)	30.290
fu mic	1.000
Active Uptake into Hepatocyte	1.000
CL R (L/h)	0.031

Table S2. Impact of Renal Impairment on Fraction Unbound (Adapted from Keller 1984, [4]).

compound	% bound HS	% bound RI	% increase (in free fraction)
azlocillin	28	25	3.00
captopril	24	18	6.00
cefazolin	84	73	11.0
cefoxitin	73	20	53.0
chloramphenicol	53	45	8.00
chlorpromazine	98	98	0.00
clonidine	30	30	0.00
n-desmethyldiazepam	98	94	4.00
desmethylimipramine	89	88	1.00
diazepam	99	94	5.00
diazoxide	92	86	6.00
dicloxacillin	96	91	5.00
diflunisal	88	56	32.0
digitoxin	97	96	1.00
digoxin	25		25.0
doxycycline	88	71	17.0
erythromycin	75	77	−2.00
etomidate	75	57	18.0
furosemide	96	94	2.00
morphine	35	31	4.00
nafcillin	88	81	7.00
naproxen	75	21	54.0
oxazepam	98	88	10.0
papaverine	97	94	3.00
penicillin G	72	36	36.0
pentobarbital	66	59	7.00
phenylbutazone	97	88	9.00
phenytoin	90	80	10.0
prazosin	95	92	3.00
prednisolone	87	88	−1.00
d-propoxyphene	76	80	−4.00
propranolol	88	89	−1.00
quinidine	88	86	2.00
salicylate	94	85	9.00
sulfamethoxazole	74	50	24.0
thiopental	72	44	28.0
triamterene	81	61	20.0
trimethoprim	70	68	2.00
d-tubocurarine	44	41	3.00
warfarin	99	98	1.00

Maximum increase in fraction unbound is highlighted in bold

Table S3. Approximation of R Values for Simulation.

Enzyme	toxin	concentration (μM)	% inhibition Reference (Barnes 2014, [1])	system	IC_{50} approximation	serum concentration (μM)	R
CYP2C9	hippuric acid	10	10	HLM			
CYP2C9.	hippuric acid	100	40	HLM	100	239 [2]	2.39
CYP3A4	indoxyl sulfate	1–10	10	HLM			
CYP3A4	indoxyl sulfate	100	35	HLM	100	135 [2].	1.35
CYP3A4	*p*-cresol	1	20	HLM		.	
CYP3A4	*p*-cresol	10	60	HLM	10	266 [3]	26.6
UGT2B7	hippuric acid	10	10	HLM			
UGT2B7	hippuric acid	100	40	HLM	100.	239 [2]	2.39
UGT2B7	*p*-cresol	10	10	HLM			
UGT2B7	*p*-cresol	100	50	HLM	100	266 [3]	2.66

Table S4. Impact of Renal Impairment on AUC of Nifedipine in Males and Females.

Population	Dose	male	female	male	female	Female AUC/Male AUC
		AUC (ng/mL·h)	AUC (ng/mL·h)	Fold increase compared to Healthy Controls		
Healthy	10	2.03E+02	2.27E+02			1.12
Healthy	30	6.30E+02	7.10E+02			1.13
Healthy	60	1.32E+03	1.49E+03			1.13
Healthy	90	2.06E+03	2.33E+03			1.13
Healthy	180	4.47E+03	5.09E+03			1.14
Sim-RenalGFR30_60	10	3.26E+02	3.50E+02	1.61	1.54	1.07
Sim-RenalGFR30_60	30	1.02E+03	1.10E+03	1.61	1.55	1.08
Sim-RenalGFR30_60	60	2.14E+03	2.32E+03	1.62	1.56	1.08
Sim-RenalGFR30_60	90	3.34E+03	3.65E+03	1.63	1.57	1.09
Sim-RenalGFR30_60	180	7.32E+03	8.06E+03	1.64	1.58	1.10
Sim-RenalGFRless_30	10	3.22E+02	3.07E+02	1.59	1.35	0.95
Sim-RenalGFRless_30	30	1.01E+03	9.62E+02	1.60	1.36	0.96
Sim-RenalGFRless_30	60	2.11E+03	2.03E+03	1.60	1.36	0.96
Sim-RenalGFRless_30	90	3.31E+03	3.20E+03	1.61	1.37	0.97
Sim-RenalGFRless_30	180	7.24E+03	7.08E+03	1.62	1.39	0.98

Table S5. Impact of CYP3A4 Expression on AUC of Nifedipine.

	Original	+2	+5	−2	−5	+2	+5	−2	−5
	AUC (ng/mL·h)					AUC original expression/AUC modified expression		AUC modified expression/AUC original expression	
Healthy	1.32E+03	5.16E+02	1.22E+02	2.99E+03	7.37E+03	2.6	11	2.3	5.6
Sim-RenalGFR30_60	2.14E+03	5.95E+02	1.45E+02	3.36E+03	8.04E+03	3.6	15	1.6	3.8
Sim-RenalGFRless_30	2.11E+03	5.41E+02	1.32E+02	3.06E+03	7.33E+03	3.9	16	1.4	3.5

Table S6. Impact of Fraction Unbound on AUC of Nifedipine for Males and Females.

Population	f_u	Male	Female	Male	Female	Female AUC/Male AUC
		AUC (ng/mL·h)	AUC (ng/mL·h)	Fold reduction in AUC when f_u is increased from 0.04 to 1.0		
Healthy	0.04	1.32E+03	1.49E+03			1.13
Healthy	0.5	9.35E+01	1.04E+02			1.11
Healthy	0.9	4.92E+01	5.47E+01			1.11
Healthy	1	4.37E+01	4.88E+01	30	31	1.12
Sim-RenalGFR30_60	0.04	2.14E+03	2.32E+03			1.08
Sim-RenalGFR30_60	0.5	1.57E+02	1.70E+02			1.08
Sim-RenalGFR30_60	0.9	8.64E+01	9.54E+01			1.10
Sim-RenalGFR30_60	1	7.76E+01	8.63E+01	28	27	1.11
Sim-RenalGFRless_30	0.04	2.11E+03	2.03E+03			0.96
Sim-RenalGFRless_30	0.5	1.64E+02	1.69E+02			1.03
Sim-RenalGFRless_30	0.9	9.39E+01	1.04E+02			1.10
Sim-RenalGFRless_30	1	8.52E+01	9.57E+01	25	21	1.12

Table S7. Impact of Changes in Fraction Unbound on Oral Clearance and Volume of Distribution of Nifedipine in Males.

**Population**	**f_u**	**CL_{po} (L/h)**	V_{ss} (L/kg)
Healthy	0.001	1.76	0.110
	0.5	862	6.48
	0.9	1.57E+03	11.7
	1	1.75E+03	13.1
Sim-RenalGFR30_60	0.001	1.10	0.120
	0.5	519	6.69
	0.9	911	11.7
	1	1.01E+03	12.9
Sim-RenalGFRless_30	0.001	1.10	0.120
	0.5	500	7.08
	0.9	844	11.9
	1	924	13.0

Table S8. Impact of Fraction Unbound on AUC of Nifedipine with Changes in CYP3A4 Expression.

		**Original**	**+2**	**+5**	**−2**	**−5**	**Original**	**+2**	**+5**	**−2**	**−5**
Population	f_u	AUC (ng/mL*h)					Fold reduction in AUC when f_u is increased from 0.04 to 1.0				
Healthy	0.04	1.32E+03	5.16E+02	1.22E+02	2.99E+03	7.37E+03					
Healthy	0.5	9.35E+01	3.65E+01	8.74E+00	2.15E+02	5.60E+02					
Healthy	0.9	4.92E+01	1.91E+01	4.58E+00	1.14E+02	3.03E+02					
Healthy	1	4.37E+01	1.70E+01	4.07E+00	1.01E+02	2.70E+02	30	30	30	29	27
Sim-RenalGFR30_60	0.04	2.14E+03	5.95E+02	1.45E+02	3.36E+03	8.04E+03					
Sim-RenalGFR30_60	0.5	1.57E+02	4.35E+01	1.07E+01	2.52E+02	6.47E+02					
Sim-RenalGFR30_60	0.9	8.64E+01	2.37E+01	5.80E+00	1.39E+02	3.66E+02					
Sim-RenalGFR30_60	1	7.76E+01	2.13E+01	5.20E+00	1.25E+02	3.30E+02	28	28	28	27	24
Sim-RenalGFRless_30	0.04	2.11E+03	5.41E+02	1.32E+02	3.06E+03	7.33E+03					
Sim-RenalGFRless_30	0.5	1.64E+02	4.16E+01	1.02E+01	2.41E+02	6.20E+02					
Sim-RenalGFRless_30	0.9	9.39E+01	2.37E+01	5.81E+00	1.39E+02	3.65E+02					
Sim-RenalGFRless_30	1	8.52E+01	2.15E+01	5.26E+00	1.26E+02	3.32E+02	25	25	25	24	22

Table S9. Impact of Competitive Inhibition on AUC of Nifedipine in Males and Females.

		**Male**	**Female**	**Male**	**Female**	
Population	R	AUC (ng/mL·h)	AUC (ng/mL·h)	Fold increase in AUC when R increases from 0 to 100		Female AUC/Male AUC
Healthy	0	1.32E+03	1.49E+03			1.13
Healthy	1	2.89E+03	3.23E+03			1.12
Healthy	10	1.29E+04	1.35E+04			1.05
Healthy	100	2.70E+04	2.61E+04	20	18	0.97
Sim-RenalGFR30_60	0	2.14E+03	2.32E+03			1.08
Sim-RenalGFR30_60	1	4.48E+03	4.87E+03			1.09
Sim-RenalGFR30_60	10	1.60E+04	1.68E+04			1.05
Sim-RenalGFR30_60	100	2.71E+04	2.72E+04	13	12	1.00
Sim-RenalGFRless_30	0	2.11E+03	2.03E+03			0.96
Sim-RenalGFRless_30	1	4.39E+03	4.25E+03			0.97
Sim-RenalGFRless_30	10	1.51E+04	1.44E+04			0.95
Sim-RenalGFRless_30	100	2.50E+04	2.31E+04	12	11	0.93

Table S10. Impact of Competitive Inhibition and CYP3A4 Expression on AUC of Nifedipine in Males.

**Fold change in CYP3A4 Expression**		**Original**	**+2**	**+5**	**−2**	**−5**	**Original**	**+2**	**+5**	**−2**	**−5**
**Population**	**R**	**AUC (ng/mL*h)**					**Fold Increase in AUC when R increases from 0 to 100**				
Healthy	0	1.32E+03	5.16E+02	1.22E+02	2.99E+03	7.37E+03					
Healthy	1	2.89E+03	1.26E+03	3.50E+02	5.90E+03	1.24E+04					
Healthy	10	1.29E+04	7.61E+03	3.04E+03	1.86E+04	2.47E+04					
Healthy	100	2.70E+04	2.39E+04	1.76E+04	2.90E+04	3.03E+04	20	46	143	9.7	4.1
Sim-RenalGFR30_60	0	2.14E+03	5.95E+02	1.45E+02	3.36E+03	8.04E+03					
Sim-RenalGFR30_60	1	4.48E+03	1.42E+03	4.03E+02	6.47E+03	1.30E+04					
Sim-RenalGFR30_60	10	1.60E+04	8.11E+03	3.33E+03	1.87E+04	2.43E+04					
Sim-RenalGFR30_60	100	2.71E+04	2.32E+04	1.74E+04	2.79E+04	2.91E+04	13	39	120	8.3	3.6
Sim-RenalGFRless_30	0	2.11E+03	5.41E+02	1.32E+02	3.06E+03	7.33E+03					
Sim-RenalGFRless_30	1	4.39E+03	1.29E+03	3.67E+02	5.89E+03	1.18E+04					
Sim-RenalGFRless_30	10	1.51E+04	7.34E+03	3.01E+03	1.71E+04	2.23E+04					
Sim-RenalGFRless_30	100	2.50E+04	2.11E+04	1.57E+04	2.55E+04	2.68E+04	12	39	119	8.3	3.7

Table S11. Impact of Fraction Unbound and Competitive Inhibition on AUC of Nifedipine.

**Population**	**f_u**	**AUC (ng/mL·h)**				**Fold Increase in AUC when R increases from 0 to 100**
		**R**				
		**0**	**1**	**10**	**100**	
Healthy	0.04	1.32E+03	2.89E+03	1.29E+04	2.70E+04	20
Healthy	0.5	9.35E+01	2.07E+02	1.06E+03	3.44E+03	37
Healthy	0.9	4.92E+01	1.09E+02	5.79E+02	2.15E+03	44
Healthy	1	4.37E+01	9.72E+01	5.18E+02	1.97E+03	45
Sim-RenalGFR30_60	0.04	2.14E+03	4.48E+03	1.60E+04	2.71E+04	13
Sim-RenalGFR30_60	0.5	1.57E+02	3.38E+02	1.52E+03	3.82E+03	24
Sim-RenalGFR30_60	0.9	8.64E+01	1.87E+02	8.85E+02	2.51E+03	29
Sim-RenalGFR30_60	1	7.76E+01	1.68E+02	8.02E+02	2.32E+03	30
Sim-RenalGFRless_30	0.04	2.11E+03	4.39E+03	1.51E+04	2.50E+04	12
Sim-RenalGFRless_30	0.5	1.64E+02	3.50E+02	1.53E+03	3.67E+03	22
Sim-RenalGFRless_30	0.9	9.39E+01	2.02E+02	9.30E+02	2.50E+03	27
Sim-RenalGFRless_30	1	8.52E+01	1.84E+02	8.51E+02	2.33E+03	27

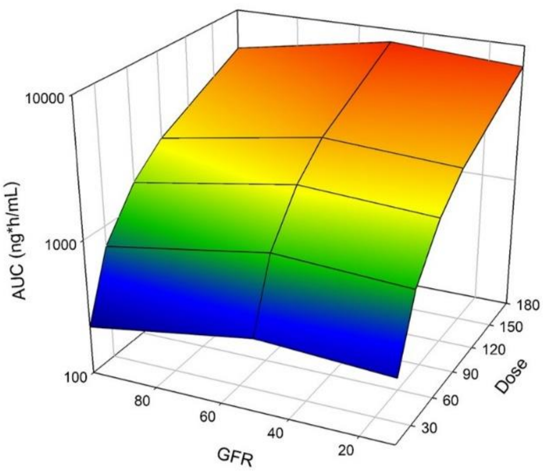

Figure S1. Impact of Renal Impairment on AUC of Nifedipine in Females. Simulations with increasing oral doses (30 to 180 mg) were administered in a fasted state to an exclusively female population. Simulations included ten trials with ten individuals in each trial. Age ranged from 21 to 65 years, and simulations were conducted with varying the degrees of RI by utilizing the following populations, Sim-Healthy Volunteers, Sim-RenalGFR_30-60, and Sim-RenalGFR_less_30 to represent 100%, 50% and 10% GFR.

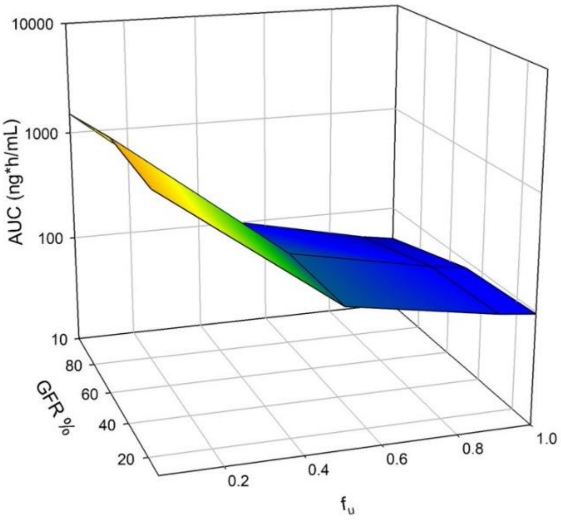

Figure S2. Impact of Fraction Unbound on AUC of Nifedipine in Females. Simulations were performed varying fraction unbound (f_u) from the observed value for nifedipine (0.04) to 1.0. A dose of 60 mg was administered orally to an exclusively female population in a fasted state. Simulations included ten trials with ten individuals in each. Age ranged from 21 to 65 years, and simulations were conducted with varying the degrees of RI by utilizing the following populations, Sim-Healthy Volunteers, Sim-RenalGFR_30-60, and Sim-RenalGFR_less_30 to represent 100%, 50% and 10% GFR.

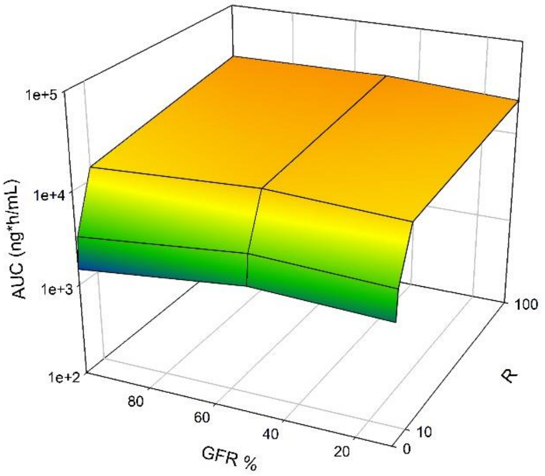

Figure S3. Impact of Competitive Inhibition on AUC of Nifedipine in Females. Simulations were performed incorporating competitive inhibition through the factor R ([I]/K_i). R was varied from 0 to 100. A dose of 60 mg was administered orally to an exclusively female population in a fasted state. Simulations included ten trials with ten individuals in each. Age ranged from 21 to 65 years, and simulations were conducted with varying the degrees of RI by utilizing the following populations, Sim-Healthy Volunteers, Sim-RenalGFR_30-60, and Sim-RenalGFR_less_30 to represent 100%, 50% and 10% GFR.

1. Barnes, K.J., et al. Inhibition of human drug-metabolising cytochrome P450 and UDP-glucuronosyltransferase enzyme activities in vitro by uremic toxins. *Eur J Clin Pharmacol*. 2014, **70**, 1097–1106.
2. Tsujimoto, M., et al. Possibility of decrease in CYP1A2 function in patients with end-stage renal disease. *Ther. Apher. Dial.* 2014, **18**, 174–180.
3. Volpe, D.A., et al. Effect of uremic serum and uremic toxins on drug metabolism in human microsomes. *Regul. Toxicol. Pharmacol.* 2014, **68**, 297–303.
4. Keller, F., et al. Pharmacokinetic effects of altered plasma protein binding of drugs in renal disease. Eur J *Drug Metab. Pharmacokinet.* 1984, **9**, 275–282.
